# A diagnostic study on the application of segmental somatosensory evoked potential examination to acquired premature ejaculation

**DOI:** 10.1093/sexmed/qfae075

**Published:** 2024-11-13

**Authors:** Yin Zhao, Minhui Chen, Jiacheng Li, Zheyang Li, Zilei Xu, Zedong Liao, Keli Xu, Xiaojun Huang

**Affiliations:** The Second Clinical Medical College, Zhejiang Chinese Medical University, HangZhou, ZheJiang, 310053, China; The Second Clinical Medical College, Zhejiang Chinese Medical University, HangZhou, ZheJiang, 310053, China; Department of Urology, The First People’s Hospital of JianDe, HangZhou, ZheJiang, 311600, China; The Second Clinical Medical College, Zhejiang Chinese Medical University, HangZhou, ZheJiang, 310053, China; The Second Clinical Medical College, Zhejiang Chinese Medical University, HangZhou, ZheJiang, 310053, China; Department of Urology, The Second Affiliated Hospital ZheJiang University School of Medicine, HangZhou, ZheJiang, 310009, China; The Second Clinical Medical College, Zhejiang Chinese Medical University, HangZhou, ZheJiang, 310053, China; Department of Urology, The First People’s Hospital of JianDe, HangZhou, ZheJiang, 311600, China

**Keywords:** acquired premature ejaculation, primary premature ejaculation, segmental dorsal penile somatosensory evoked potential, neuroelectrophysiology, diagnosis

## Abstract

**Background:**

Premature ejaculation (PE), affecting approximate 5%, has an unclear pathogenesis, limited treatment efficacy, and a lack of effective diagnostic methods.

**Aim:**

This prospective diagnostic study aimed to compare segmental dorsal penile nerve somatosensory evoked potentials (DNSEP) differences among patients with acquired premature ejaculation (APE), primary premature ejaculation (PPE), and healthy controls.

**Method:**

This prospective diagnostic study examined patients suffering from PE who visited the outpatient clinic of the Department of Urology of the Second Affiliated Hospital of Zhejiang University School of Medicine from January 1, 2022 to February 28, 2023. According to the definition of PE by the ISSM, 16 cases comprised the healthy control group, 31 in the APE group, and 28 in the PPE group. Each group was examined based on the segmental DNSEP with electrodes recording at multiple locations (the selected location was at the Cz and the C7). The latency time of the evoked potential obtained at Cz was P40, and that obtained at C7 was P30. The P30/P40 ratios of P40, P30, and DNSEP wave amplitudes at C7 and Cz were compared among the 3 groups of patients.

**Result:**

No group differences were found in P40 latency at Cz. However, PPE showed higher DNSEP amplitude at Cz, while APE showed lower amplitudes compared with controls. Both APE and PPE had significantly shorter P30 latency at C7 than controls. SEP amplitude at C7 was similar in APE and PPE but lower than in controls. The P30/P40 ratio was lower in APE compared with PPE and controls.

**Clinical implications:**

Segmental SEP may offer more assistance in localizing neurological lesions, potentially guiding clinical treatment.

**Strengths and limitations:**

In this study, the innovative use of the P30/P40 ratio was proposed, maintaining consistency in emotional states and measurement conditions for the same patient. However, limitations include a restricted number of patient cases and challenges in obtaining a diverse control group, potentially introducing bias. In addition, not considering subclinical premature ejaculation and the comorbidity of PE + ED (LCEE) in patient stratification is another limitation of this study. Results suggest a correlation between secondary PE and underlying conditions, such as obesity and lumbar spine injuries. The study validates multi-site somatosensory-evoked potential examination for locating neural lesions but acknowledges the need for future invasive needle electrode AEP testing to analyze neuropathological changes more comprehensively.

**Conclusion:**

In conclusion, segmental DNSEP examination aids in localizing neuropathy in APE patients, and the P30/P40 ratio proves more accurate in diagnosing APE than P40 alone.

## Introduction

A correlation has been established between the development of acquired premature ejaculation (APE) and the diseases, such as diabetes mellitus,^1^ obesity,^2^ prostatitis, hypertension,^3^ and lumbar spine injuries^4^ that may cause damage to peripheral and spinal cord nerves. Somatosensory evoked potential (SEP) is defined as the bioelectrical activity generated by the nervous system after sensing various specific stimuli outside the body; it reflects the functional integrity of various conduction pathways in the nervous system.^5^ Segmental SEP can be used in clinical practice to analyze the lesion sites in nerve afferent pathways. The waveform of evoked potentials is composed of a series of positive or negative waves. The upward waves are represented by N, indicating negative phases, while the downward waves are represented by P, indicating positive phases. The latency of each wave represents the transmission time required for the nerve impulse to travel from the stimulation site to the source of the wave peak, depending on the length of the sensory pathway, the number of synapses, and the nerve conduction velocity. The latency of a waveform is usually represented as “peak direction+latency.”^6^ For example, in the upper limb SEP examination, when stimulating the median nerve, a waveform with a downward peak is obtained through the recording electrode at the central vertex (Cz) after about 13 ms, which is named P13. For example, Koutlidis et al.^7^ evaluated chronic inflammatory demyelinating polyneuropathy and other sensory neuropathies by segmental SEP. Polo et al.^8^ explored the root cause of upper limb muscle atrophy in adolescents using segmental SEP. For several years, neuropathy in patients with PE has been assessed by dorsal penile nerve somatosensory evoked potentials (DNSEP), utilizing the head as the recording electrode; however, segmental DNSEP has not yet been used in the clinical setting. Does segmental DNSEP examination have a different diagnostic value for APE, primary premature ejaculation (PPE), and healthy controls?

## Background

It is widely accepted that PE is the most common sexual dysfunction disease, with a prevalence of 22.7%-31%.^9^ However, according to the ISSM definition, the incidence rate of clinical premature ejaculation is approximately 5%.^10^ In 20 years, somatic and neurobiological hypotheses of the etiology of PE have been established. Currently, multiple biological factors associated with PE include central 5-HT neurotransmitter disorders, hyperexcitability of the sympathetic nervous system, penile hypersensitivity, hypertension, diabetes mellitus, erectile dysfunction (ED), spinal cord injuries, chronic prostatitis, and other disorders.^11^ Since the mechanisms underlying the pathogenesis of PE are not yet clarified, the current efficacy of the treatment is limited. Therefore, improving the diagnosis of PE and developing safe and effective medications are urgent clinical issues.

The diagnosis of PE currently relies mainly on patient history collection, intravaginal ejaculation latency time (IELT), and premature ejaculation diagnostic tool (PEDT).^10^^,^^12^ Among these, IELT is extensively utilized in clinical trials and observational studies for the assessment of PE. According to the ISSM definition of PE in 2014,^10^ (1) ejaculation consistently or frequently occurs within 1 min of vaginal penetration (PPE) or the latency time of ejaculation is significantly shortened, usually <3 min (APE). (2) Inability to control ejaculation. (3) Negative personal emotions and behaviors, including distress, frustration, and/or avoidance of sexual intimacy. Although an objective measure, IELT requires the patient’s partner to use a stopwatch during sexual activity, which might compromise the sexual pleasure for both the patient and their partner.^10^ DNSEP is an objective indicator for the diagnosis of PE and has been gaining attention.^13^ Sun et al.^14^ used DNSEP with Cz as the placement point for recording electrode and showed that patients with APE might have nerve damage that affects the results of SEP examination. The mechanism and site of the pathological changes in this nerve injury require further investigation. Moreover, relying solely on patient history collection and physical examination to assess nerve damage may lead to errors and omissions and is limited to speculation. Although computed tomography and magnetic resonance imaging can reveal morphological changes, they may not reflect the functional state of the spinal cord accurately, especially since neuropathy in APE is often early damage caused by chronic diseases and hence, may not be evident in morphological changes. Thus, studying the mechanisms underlying these changes would offer new insights for the diagnosis of APE.

## Methods

### Participants

This study enrolled patients with PE from the male urology outpatient department and healthy males undergoing physical examinations at the Second Affiliated Hospital of Zhejiang University School of Medicine. Participants were classified into 3 groups: (i) PPE group, comprising adult heterosexual males with a stable sexual partner, regular sexual activities, IELT <1 min on the first sexual intercourse, a PEDT score ≥11, and normal levels of sex hormones. (ii) APE group, including adult heterosexual males with a stable sexual partner, regular sexual activity, IELT <3 min during sexual intercourse, which occurred suddenly or gradually, and a PEDT score ≥11. (iii) Healthy control group, consisting of adult heterosexual males with a stable sexual partner, regular sexual activity, and IELT >3 min. The exclusion criteria were as follows: those with severe psychological and mental disorders, severe underlying health conditions, external genital deformities, ED, currently undergoing medication treatment or discontinuing medication within 1 week, and inability to undergo examination.

Based on the above inclusion and exclusion criteria, participants were assigned to the PPE group (28 cases), the APE group (31 cases), and the healthy control group (16 cases). The study was approved by the Ethics Committee of the hospital (IRB2022-0264), and all participants provided written informed consent.

### Instrument

The Nicolet EDX electromyography/evoked potential instrument (Natus, United States) was used for the DNSEP examination in this study.

### Procedure

**Figure 1:**
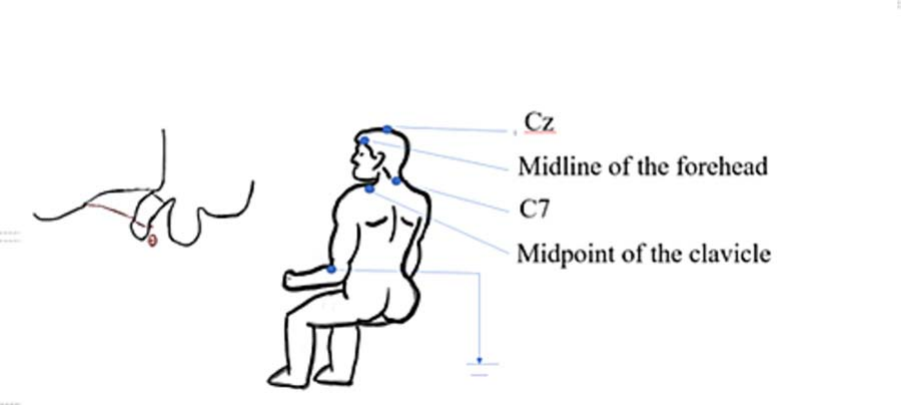
Schematic diagram of dorsal penile nerve somatosensory evoked potentials.

DNSEP examination method: The patient is placed in a supine position, and the penis as well as the skin of the head and neck is exposed. The examination is initiated by wiping the penis with a 75% alcohol cotton swab, The circular anode electrode is placed 1 cm posterior to the coronal sulcus of the penis. The cathode is positioned 2 cm away from the anode electrode on the shaft of the penis. The ground electrode is affixed between the recording and stimulating electrodes (on the right wrist). The head recording and reference electrodes were placed 2 cm posterior to Cz and at the midline of the forehead (FPz), respectively (Schematic diagram is shown in Figure 1). The electrode impedance is adjusted to be less than 5 kΩ. The penile sensation threshold of the patient is measured. The current intensity is gradually increased from 0 mA until the patient feels a pinprick-like sensation in the penis, which is the penile sensation threshold. Subsequently, the current is adjusted to 3 times the sensation threshold, with a frequency of 3 Hz and a pulse duration of 1 ms. The stimulation is repeated 200 times, and the latency and wave amplitude are recorded. Then, the head electrode is removed, and the neck recording and reference electrodes are placed at the spinous process of the seventh cervical vertebra (C7) and the midpoint of the right clavicle, respectively. The same method of stimulation was applied to the penis, and the latency and wave amplitude were recorded again.

### Data analysis

Statistical analysis was performed using Solutions Statistical Package for the Social Sciences 26.0 (SPSS Inc., Chicago, IL, United States). Normally distributed measurement data were expressed as arithmetic mean ± standard deviation, while non-normally distributed measurement data were expressed as median (interquartile range). Among the data, the IELT exhibits a positive skew and contains notably pronounced outliers. Consequently, it is more appropriately represented as the geometric mean ± geometric standard deviation.^15^ One-way ANOVA was used to compare the data among the 3 groups. The least significant difference (LSD) method was applied for post hoc multiple comparisons of data with homogeneity of variance, while the Games–Howell method was used for data with the heterogeneity of variance. The chi-square test was used for nominal data comparison. *P* < 0.05 indicated statistically significant differences.

## Results

### Demographic data

Demographic data (age, height, weight, IIEF-5 score, testosterone, estradiol, IELT, PEDT) were compared among the 3 groups. No statistically significant differences were observed in age, height, weight, IIEF-5 score, testosterone, or estradiol among the three groups (shown as Table 1).

**Table 1:** General information.

Groups	PPE (*n* = 28)	APE (*n* = 31)	Healthy control (*n* = 16)	*P*-values
Age (years)	31 ± 5.4	30.1 ± 7	29.8 ± 5.7	0.721
Height (cm)	172.1 ± 5.6	170.1 ± 5.4	169.7 ± 6	0.272
Weight (kg)	69 ± 7.3	72.4 ± 7.8	67.7 ± 4.2	0.057
Testosterone (nmol/L)	20.0 ± 4.1	19.9 ± 1.8	20.6 ± 2.3	0.722
Estradiol (pg/mL)	27.6 ± 5.2	27.0 ± 6.1	30.6 ± 4.7	0.104
IELT (s)	33.67 ± 1.27	32.75 ± 1.27	782.71 ± 1.31	0.000^*^
PEDT	12.8 ± 2.6	13.3 ± 2.5	5.4 ± 1.9	0.000^*^
IIEF-5	22.7 ± 1.3	22.7 ± 1.0	22.7 ± 1.0	0.958

### Comparison of clinical symptom scale between patients with PE and healthy controls

The DNSEP latency time and wave amplitude, termed P40 (central vertex) and P30 (the seventh cervical vertebra), estimated PSSR time and penile cutaneous perception threshold, respectively, for the 3 groups (shown as Table 2).

**Table 2:** Comparison of electrophysiologic results of penile in the 3 groups.

Groups	PPE (*N* = 28)	APE (*N* = 31)	Healthy control (*N* = 16)	*P*-values
C7 latency time (ms)	30.78(5.46)^a^	31.1(6.0)^a^^,^^c^	37.75(11.18)	0.004^*^
C7 wave amplitude (μA)	1.65(0.68)^a^	1.6(1.09)^a^^,^^c^	2.7(0.95)	0.000^*^
Cz latency time (ms)	37.48 ± 5.05^a^	39.13 ± 4.91^b^^,^^c^	40.69 ± 5.92	0.058
Cz wave amplitude (μA)	1.6 ± 0.48^a^	0.87 ± 0.34^a^^,^^d^	1.34 ± 0.38	0.001^*^
Cz shortened headcount	16^a^	11^a^^,^^d^	4	0.001^*^
PSSR (ms)	399.9 (446.9)^a^	409.4 (93.8)^a^^,^^c^	1519.7 (516.1)	0.001^*^
P30/P40	0.86 ± 0.09^b^	0.79 ± 0.09^a^^,^^d^	0.91 ± 0.05	0.001^*^
Diminished penile cutaneous perception threshold headcount	19^a^	23^a^^,^^d^	1	0.001^*^

a Indicates comparison with healthy control group, *P* < 0.05

b Indicates comparison with healthy control group, *P* > 0.05

c Indicates comparison with PPE group, *P* > 0.05

d Indicates comparison with PPE group, *P* < 0.05

Note: C7 refers to the placement of recording electrodes for SEP examination at the projected point on the body surface of the seventh cervical vertebra spinous process; Cz refers to the placement of recording electrodes on the skin at the Cz for SEP examination; P30 refers to the SEP latency time obtained at C7; P40 refers to the SEP latency time obtained at Cz.

Comparisons were made among the 3 groups of data. No significant difference was observed in the DNSEP latency time P40 obtained at Cz among the 3 groups (*P* > 0.05). DNSEP wave amplitude at Cz showed significant differences among the 3 groups (*P* < 0.05), with the PPE group (1.6 ± 0.48) significantly higher than the healthy control group (1.34 ± 0.38) and the APE group (0.87 ± 0.34) significantly lower than the healthy control group.

SEP latency time P30 obtained at C7 did not show a statistically significant difference between the PPE (30.78 ± 5.46) and APE (31.1 ± 6.0) groups (*P* > 0.05). The PPE and APE groups were significantly lower (*P* < 0.05) than the healthy control group (37.75 ± 11.18). SEP wave amplitude at C7 did not show any significant difference between the PPE group (1.65 ± 0.68) and the APE group (1.6 ± 1.09) (*P* > 0.05). However, both groups showed significantly lower values than the healthy control group (*P* < 0.05).

P30/P40 showed significant differences in the APE group (0.79 ± 0.09) compared with the PPE (0.86 ± 0.09) and healthy control (0.91 ± 0.05) groups (*P* < 0.05). On the other hand, no statistically significant difference was detected between the PPE and healthy control groups (*P* > 0.05), and the P30/P40 was lower in the APE group than in the PPE and healthy control groups.

**Figure 2:**
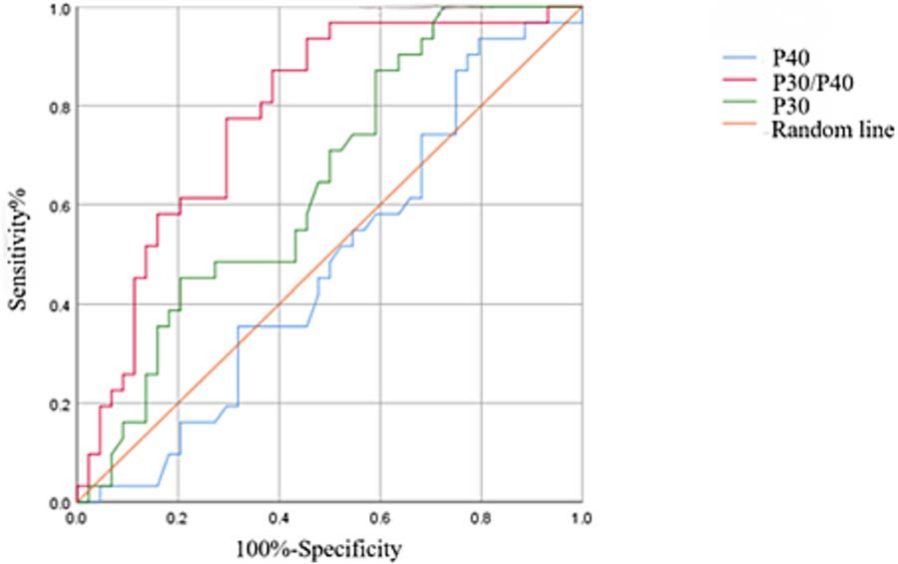
ROC diagnostic curve: The proximity of the curve to the upper left corner indicates high accuracy.

The current data revealed that 71% of APE patients had a P30/P40 ratio lower than the healthy control group, with a higher sensitivity, consistent with previous penis skin sensitivity examinations (Table 3). The receiver operating characteristic (ROC) diagnostic curve was used to validate the diagnostic efficiency of P30 (shown at C7 in Figure 2), P40, and the P30/P40 ratio for the diagnosis of APE. As shown in the ROC curve in Figure 2, the diagnostic advantage of P30/P40 in patients with APE is superior to the observation of P40 or P30 alone. The ROC curve represents the ability of a specific diagnostic method, which is a comprehensive index, to distinguish between samples of specific patient groups and non-patient groups. Sensitivity (Se), also known as the true positive rate, represents the percentage of people who actually have the condition and are correctly diagnosed as patients according to the diagnostic test standard. It reflects the ability of the diagnostic test to identify the patient. Specificity (Sp), also known as the true negative rate, represents the percentage of people who actually do not have the condition and are also correctly diagnosed as healthy individuals according to the diagnostic test standard. It reflects the ability of the diagnostic test to identify healthy individuals. The area under the ROC curve (AUC) is the area surrounded by the ROC curve, the x-axis, and the points (1,0) and (1,1). The closer the AUC is to 1 and the closer it is to the point (0,1), the better the authenticity of the diagnostic test.

**Table 3:** P30/P40 Ratio Test Results for Distinguishing AEP and Non-AEP Groups.

Actual situation	Test result		Total
	P30/P40 <0.86^*^	P30/P40 >0.86	
AEP group	26	5	31
Non-AEP group	17	27	44
Total	43	32	75

^^*^^0.86 denotes the optimal cutoff value for distinguishing APE using P30/P40

The analysis demonstrated that the respective areas under the receiver operating characteristic curves (AUCs) for P40, P30, and the P30/P40 ratio were 0.482, 0.646, and 0.782. With a positive predictive value of 60% for the P30/P40 ratio, it is evident that this combined metric offers superior diagnostic efficiency in identifying APE compared with the use of P30 or P40 individually (Table 3).

## Discussion

### Individual observation of P30 and P40 for PE diagnosis

The data from this study (Table 1) showed that there is no significant difference in general information between the APE group, the PPE group and and healthy control groups. Moreover, the data from this study (Table 2) indicated that there were no significant differences in P40 among PPE (37.48 ± 5.05 ms), APE (39.13 ± 4.91 ms), and healthy control groups (40.69 ± 5.92 ms). This finding contradicted the results reported in the literature, suggesting that the SEP latency in PE patients is significantly shorter than that in the healthy control group.^12^ This finding indicated that the majority of patients with PE included in this study did not have fast conduction in peripheral nerves. However, 67.86% of patients in the PPE group (*n* = 28) had decreased penis skin sensory thresholds, and 74.19% of patients in the APE group (*n* = 31) had decreased penis sensory thresholds, indicating peripheral nerve sensitization in these patients but could not be detected by conventional DNSEP examination. The lack of significant P40 shortening in most APE patients might be due to factors such as anxiety and nervousness in patients during the examination, variation in skin conductivity at the testing site, and improper adjustment of skin electrode impedance by the operator during the procedure.^16^ Head DNSEP detection alone has limited sensitivity in detecting PE, and it is also influenced by many practical factors during the examination.

In this study, significant differences were detected in P30 among the APE group (31.1 ± 6.0 ms), the PPE group (30.78 ± 5.46 ms), and the healthy control group (37.75 ± 11.18 ms). P30 in the PE groups was significantly shorter than in the healthy control group, and 93% of SEP patients had P30 values lower than the median of the healthy control group, while 82% of PPE patients had P30 values lower than the median of the healthy control group. This finding suggested that in most PE patients, the nerve conduction velocity from the dorsal nerve of the penis to the C7 site is significantly faster than in the normal control group; this conclusion corresponds to the results obtained from glans sensory threshold measurements. Thus, P30 is deemed to have a higher sensitivity for PE diagnosis than P40. However, P30 obtained in this study has a high degree of dispersion that did not pass the variance chi-square test, making it difficult to determine a reasonable standardized value to guide clinical diagnosis. Thus further processing of patient data is essential.

### P30/P40 ratio interpretation

This study innovatively proposes the ratio of P30/P40. Since the P30 and P40 of the same patient were measured within a short interval, the patient’s emotions and mental states were consistent, and the operator’s technique, steps, and parameter adjustments during the procedure could also be maintained at the same level. Thus, it could be speculated that under this data processing method, factors such as patient weight, height, and individual differences that affect the overall results might be offset by dividing P30 by P40. Therefore, the effects of local factors on peripheral nerves might be accentuated.

The P30/P40 ratio passed the test for variance homogeneity, indicating normal distribution data. Significant differences were observed between the PPE (P30/P40: 0.86 ± 0.09) and APE (P30/P40: 0.79 ± 0.09) groups (*P* < 0.001), as well as between the APE and healthy control (P30/P40: 0.91 ± 0.05) groups (*P* < 0.001), while no significant differences were observed between the PPE and healthy control groups. The P30/P40 ratio of the APE group was smaller than that of the PPE and healthy control groups. The results showed that the AUC for P40, P30, and P30/P40 was 0.482, 0.646, and 0.782, respectively, indicating that P30/P40 has a higher diagnostic efficiency in diagnosing APE compared with P30 or P40 alone. In conclusion, the application of SEP is feasible for PE diagnosis. In the clinical setting, segmental SEP was used to diagnose APE, and the value of P30/P40 was more efficient in the diagnosis of APE patients than either P30 or P40. Therefore, the localization function of segmental SEP for neuropathy might be valuable in guiding clinical treatment.

Furthermore, based on the results of P30/P40, it can be inferred that P30/P40 in APE patients is significantly lower than that in PPE patients and healthy controls, suggesting that the majority of the accelerated neural transmission in APE patients may occur in a specific segment of the neural pathway rather than accelerating the entire pathway. This phenomenon leads to a significant difference in P40 between APE patients and PPE/healthy control groups.

### Neural sensitization and PE

This study located neural lesions in the sensory pathways of APE patients, leading to accelerated neural conduction in certain segments. These lesions may be caused by some primary underlying conditions in the patients. Physical neural damage usually involves the disruption of the nerve sheath, and the demyelinating lesions in afferent nerves decelerate the neural signal conduction. In PE patients, especially in those with APE, the neural lesions are manifested as accelerated conduction in neurophysiological studies, which cannot be explained solely by the theory of neural injury. Some studies have suggested another pathophysiological change termed neural sensitization.^17^

Interestingly, neural sensitization is commonly observed in patients with chronic, refractory pain, often associated with long-term stimulating factors. For example, the somatosensory pathway is activated in chronic pain caused by lumbar disc herniation. In addition to peripheral inflammatory injuries, the central nervous system also undergoes a series of complex changes. Central sensitization is the primary cause of these changes and the main pathological basis of pain hypersensitivity. The International Association for the Study of Pain defines central sensitization as an increased responsiveness of nociceptive neurons in the central nervous system to their normal or subthreshold afferent input.^14^

The mechanism of pathological pain sensitization has not yet been fully elucidated. The widely accepted theoretical mechanisms include peripheral and central sensitization after neural injury. Factors leading to peripheral sensitization include sympathetic sprouting causes ectopic discharge activities in altered ion channels, peripheral inflammatory responses, noncoding RNA-related gene transcription disorders, and changes in pain signal transmission. Factors inducing central sensitization include the corresponding inflammatory responses, abnormal activation of glial cells, and functional disorders in the central nervous system.^18^

In some APE patients, the P30 in C7 is significantly shortened compared with the normal control group, while the overall P40 does not show a significant shortening. Thus, it can be speculated that multiple factors contribute to the lesions in these patients. Neural sensitization is detected in the peripheral and low-level central spinal pathways, and neural damage decelerates neural conduction.

Among the subjects in this experiment, 83% of APE patients had primary diseases, including diabetes mellitus, obesity, prostatitis, hypertension, lumbar spine injuries, and other factors that could lead to peripheral or spinal nerve damage, indicating a correlation with PE. Some studies have shown that these primary diseases are the common causes of pain hypersensitivity, which is consistent with the results of this study. Meanwhile，the study reveals that the use of the P30/P40 ratio in diagnosing APE is more efficient than using P30 or P40 alone, which may significantly enhance diagnostic accuracy and therapeutic outcomes.

Identifying specific variations in neural conduction velocity among APE patients enables physicians to devise personalized treatment plans, potentially including interventions targeting specific neural pathways. Traditional diagnostic methods, such as IELT, can be subject to various influences. In contrast, DNSEP offers a more objective supplementary diagnostic approach. The findings not only deepen the understanding of the underlying pathophysiological mechanisms of APE, such as neural sensitization, but also lay the groundwork for the development of innovative treatment strategies. Achieving more precise diagnostics and targeted treatments is expected to substantially improve patients’ sexual function and overall quality of life. Furthermore, the research results point the way for future studies, including in-depth exploration of the relationship between underlying diseases and APE, as well as more detailed analysis of potential neuropathological changes.

### Deficiencies and limitations

1. The number of patients included in the study is limited, especially the difficulty in collecting subjects for the healthy control group, and the single type of subjects may introduce some bias in data collection and analysis.
2. Based on the experimental results, it was hypothesized that APE is associated with the presence of primary diseases, such as obesity, prostatitis, lumbar spine injuries, and other factors that may cause peripheral or spinal nerve damage, suggesting a correlation between such factors and APE. Further review after the improvement of these primary causes could make a convincing argument.
3. Due to the limitations of patient compliance and ethical approval, the SEP detection at the lumbar region (T12-L1) was abandoned in this study. Using needle electrodes for neural signal detection at the corresponding site in SEP examination can eliminate interference. This study validated the efficiency of multisite SEP detection for the localization of neural lesions in APE. Alternatively, further invasive needle electrode SEP examination and refined analysis of neural pathology may be conducted.
4. Colonnello^19^ pointed out that PE can be divided into types such as clinical premature ejaculation, subclinical premature ejaculation (SPE), erectile and ejaculatory dyscontrol. In this paper, we only discussed clinical premature ejaculation such as PPE and APE, without considering SPE and the comorbid condition of PE and ED (LCEE). This may lead to the research results being unable to fully reflect the conditions of all PE patients. We will consider incorporating these factors in future research for a more comprehensive analysis.

## Conclusion

The application of segmental SEP in PE diagnosis is feasible. Segmental SEP examination can locate neural lesions in patients with APE. The diagnostic efficiency of APE using P30/P40 value is higher than P40 alone. This hypothesis is grounded in the observed differences in neural conduction velocities and the potential for localized neural sensitization in APE patients, which the P30/P40 ratio may more effectively capture.
